# Innocent Amusement

**Published:** 1872-07

**Authors:** 


INNOCENT AMUSEMENT.—We are informed that a sportingly-inclined gentleman of this city, is amusing himself by firing his revolver at numerous and aggressive serpents, which intrude themselves upon his society, while he is confined to his house by sickness. It is said that he has made many capital hits, and that his neighbors are having their houses iron-clad, for prudential reasons.

